# Reference values for intake of six types of soluble and insoluble fibre in healthy UK inhabitants based on the UK Biobank data

**DOI:** 10.1017/S1368980021002524

**Published:** 2022-05

**Authors:** Artem Shevlyakov, Dimitri Nikogosov, Leigh-Ann Stewart, Miguel Toribio-Mateas

**Affiliations:** 1 Atlas Biomed Group Limited, Tower Bridge House, St. Katharines Way, London E1W 1DD, UK; 2 School of Health and Education, Middlesex University, The Burroughs, London, UK; 3 London, School of Applied Sciences, London South Bank University, London, UK

**Keywords:** Nutrition, Dietary fibre, Prebiotics, Gut microbiota, UK Biobank, Reference values, Dietology

## Abstract

**Objective:**

To obtain a set of reference values for the intake of different types of dietary fibre in a healthy UK population.

**Design:**

This descriptive cross-sectional study used the UK Biobank data to estimate the dietary patterns of healthy individuals. Data on fibre content in different foods were used to calculate the reference values which were then calibrated using real-world data on total fibre intake.

**Setting:**

UK Biobank is a prospective cohort study of over 500 000 individuals from across the United Kingdom with the participants aged between 40 and 69 years.

**Participants:**

UK Biobank contains information on over 500 000 participants. This study was performed using the data on 19 990 individuals (6941 men, 13 049 women) who passed stringent quality control and filtering procedures and had reported above-zero intake of the analysed foods.

**Results:**

A set of reference values for the intake of six different types of soluble and insoluble fibres (cellulose, hemicelluloses, pectin and lignin), including the corresponding totals, was developed and calibrated using real-world data.

**Conclusions:**

To our knowledge, this is the first study to establish specific reference values for the intake of different types of dietary fibre. It is well known that effects exerted by different types of fibre both directly and through modulation of microbiota are numerous. Conceivably, a deficit or excess intake of specific types of dietary fibre may detrimentally affect human health. Filling this knowledge gap opens new avenues for research in discussion in studies of nutrition and microbiota and offers valuable tools for practitioners worldwide.

The term ‘dietary fibre’ refers to a diverse group of organic compounds found in edible plants. The exact definition has changed since its first appearance in scientific press^(1)^. Codex Alimentarius, a WHO-approved guideline to nutrition labelling, defines dietary fibre as ‘carbohydrate polymers with ten or more monomeric units, which are not hydrolysed by the endogenous enzymes in the small intestine of humans’^(2)^. Indigestibility by endogenous human enzymes is a core property of dietary fibres.

Foods high in fibre are less energy-dense, and indigestible fibre might inhibit the absorption of high-calorie nutrients^(3–5)^, directly affecting food properties and human health. In the early 1970s, Heaton proposed that dietary fibre may decrease the energy/satiety ratio of food and that adding dietary fibre to diet may help combat obesity^(6)^. Research confirmed that dietary fibre promotes weight control and maintains healthy levels of metabolic markers in humans^(7–12)^. This is intriguing, considering the steady increase in the prevalence of overweight and obesity^(13–16)^ – risk factors of metabolic syndrome^(17)^, type 2 diabetes^(18,19)^, CVD^(20,21)^ and cancer^(22–25)^.

Gut flora plays a complex role in health and disease. Tens of trillions of commensal, symbiotic and pathogenic microorganisms inhabit the human gastrointestinal system^(26,27)^, their diversity and quantities increasing from stomach to small intestine to colon^(28,29)^. Although often used interchangeably, microbiota is the term that refers to community of microorganisms themselves, while the collective microbial genomes are known as the microbiome^(30)^.

Dysbiosis (a combination of unfavourable changes in the composition of gut microbiota) may contribute to the development of metabolic and immune disorders such as ulcerative colitis^(31)^, Crohn’s disease^(32)^, type 2 diabetes and obesity^(33,34)^, and liver cirrhosis^(35)^. It may also be associated with cognitive function and mental health conditions^(36,37)^. Maintaining a sufficient population and diversity of microbiota seems crucial for human health.

As a main source of microbial nutrition, fibre contributes to the overall health and well-being. Dietary interventions may lead to changes in microbiota composition: the gut microbiota responds by altering fermentation, composition and colony sizes^(38)^. Diets rich in certain types of fibre seem to be better at promoting the growth of beneficial bacterial populations; different bacterial genera seem to thrive on different types of nutrients.

While some fibres, such as inulin, are widely acknowledged for their ability to induce beneficial modifications in gut microbiota composition and function^(39–41)^, other types of fibre may also exert positive influence through less understood pathways. It is plausible that low fibre diversity (i.e. excess intake or deficiency of a certain type of fibre) may shift the composition of the gut microbiota, promoting excessive growth of specific bacteria while not supporting other ones^(41,42)^.

According to a study published in 2010, up to 80 % of clinical decisions are based on an interpretation of laboratory results^(43)^. Reference ranges for the overwhelming majority of biochemically important nutrients have been developed and are widely applied. Yet there are no guidelines which take into account the diversity of fibre types and no reference values for intake of different types of fibre. Clearly, obtained test values are unusable unless it is known what reference they should be compared to.

Little to no research has been done to quantify and standardise the recommended daily intake of specific types of fibre. This research aims to make a step towards filling this gap. By calculating the reference values for the intake of specific types of fibre, we hope to expand the small pool of information, promoting further research and supporting more solid dietary and clinical decisions.

## Materials and Methods

Establishing the reference values for dietary intake requires a large sample representative of the whole population, and a method of collecting detailed information. For the UK population, such a database is provided by the UK Biobank, a prospective cohort study of over 500 000 individuals from across the United Kingdom. Participants aged between 40 and 69 years were invited to one of twenty-two centres across the UK between 2006 and 2010. Blood, urine and saliva samples were collected, physical measurements were taken, and each individual answered an extensive questionnaire about health and lifestyle. Full UK Biobank protocol and rationale are available online^(44)^. For this study, data access to UK Biobank was granted under application #36183.

Dietary intake of the UK Biobank participants was evaluated with Oxford WebQ tool, a validated web-based questionnaire which assesses the 24-h intake of 206 foods and 32 beverages^(45)^. This evaluation was performed several times, none of which were mandatory. These data, grouped into the ‘Diet by 24-hour recall’ (Category 100090) section of the UK Biobank, were used to estimate the amount and type of food consumed by the subjects of the study.

Unfortunately, these data do not contain a detailed breakdown of fibre types. Data on content of fibre by type were taken from the article by Marlett and Cheung^(46)^, reporting the content of two different soluble and four different insoluble types of fibre, as well as the relevant totals, for 228 various foods. We used the provided serving weights to calculate the content of fibre per 100 g of food.

The fibres used in the analysis are summarised in Table 1 (the classification of fibre as ‘soluble’ or ‘insoluble’ corresponds to the original article) ^(46)^. For total soluble, total insoluble and overall total fibre content, we used the data from the article^(46)^; total pectin and total hemicelluloses fibre content were calculated manually.

**Table 1:** Types of fibre used in the current analysis^(46)^

Soluble	Insoluble
Hemicelluloses	Cellulose
Pectin	Hemicelluloses
	Pectin
	Lignin

The Oxford WebQ questionnaire reports food consumption in servings. To convert servings to grams and calculate the net content of specific nutrients, we used the Food Portion Sizes book compiled by the Food Standards Agency (widely known as the Maff Handbook) ^(47)^, which had been used during the preparation of the Oxford WebQ^(45)^. This study used the 3rd edition for reference, as the 2nd edition (used for the Oxford WebQ) is out of print and inaccessible.

The foods reported in the Maff Handbook^(47)^ do not precisely match the foods from the Oxford WebQ. We performed the mapping by hand, discarding several food items from the Oxford WebQ that were not present in the Maff Handbook^(47)^. In multiple cases, the matches were ambiguous due to the nature of reporting in the Maff Handbook^(47)^ and low specificity of some of the UK Biobank foods. For example, a UK Biobank question *‘How many slices of sliced bread did you eat yesterday?’*, matched to several items from the Maff Handbook^(47)^ depending on the type of bread and the thickness of the slice. In such cases, the weight from the ‘average’ portion was selected. Full mappings are presented in Supplementary Table 1.

Oxford WebQ uses an open-ended system for the number of consumed servings. Prior to calculating individual food intake, the answers were converted to remove ambiguity, turning 3+, 4+, 5+ and 6+ servings into 3, 4, 5 and 6 servings, respectively. ‘Less than 1’ serving was considered as 0·5 of the serving.

The foods analysed in the Marlett and Cheung article^(46)^ also did not directly correspond to the foods reported either in the UK Biobank or the Maff Handbook^(47)^. We developed a tagging system and assigned from one to five tags to each food in the Marlett and Cheung article^(46)^. We then calculated the fibre content for each tag as the average fibre content per 100 g of each food with this tag. Full mapping of tags is presented in Supplementary Table 2.

All the foods present in the UK Biobank were also tagged as represented in Supplementary Table 3. The calculated per-tag fibre values were mapped to the Oxford WebQ items. We used the average of the values if the food had been assigned two or more tags and discarded the foods with no tags. After the labelling, 113 foods from the UK Biobank questionnaire remained, with fibre content per 100 g and weight of serving available for each. The breakdown of the fibre content per 100 g of each analysed food is presented in Supplementary Table 4.

We performed a quality check of the UK Biobank data to exclude unreliable and incomplete entries. The included participants had to meet all of the following criteria:

- Data on genetic sex (Data-Field 22001), self-reported ethnic background (Data-Field 21000), BMI (Data-Field 21001) and self-reported medical history (Data-Fields 20001 and 20002) were available;
- Self-reported sex (Data-Field 31) matched genetic sex (Data-Field 22001) and was consistent between visits;
- Self-reported ethnic background (Data-Field 21000) was either British, Irish or Other White and was consistent between visits;
- BMI (Data-Field 21001) and weight (Data-Field 21002) had been measured at least once each;
- Participant had reported consuming at least one of the foods that had successfully been mapped to detailed fibre content.

After filtering, information on the consumption of 113 foods by 196 608 participants (88 626 males and 107 982 females) remained. Of these, we kept only the individuals who deemed themselves healthy and reported no diseases at any of the visits (Data-Fields 22001 and 22002 contained no entries) and had BMI within normal range of (18·5, 25).

For each of the selected participants, the intake of specific types of fibre, as well as total intake of fibre, were calculated as follows:




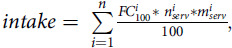



where *n* – total number of reportedly consumed food items containing the analysed type of fibre; *i* – the number of reportedly consumed Oxford WebQ food; *FC*_100_^*i*^ – calculated fibre content in 100 g of food *i*; *n*_serv_^*i*^ – number of consumed servings of food *i*; and *m*_serv_^*i*^ – mass of a single serving of food *i* (according to^(47)^). Total daily intake of fibre was calculated by adding up the total fibre content of each consumed food provided in the Marlett and Cheung article^(46)^. The BMI and weight of each participant were calculated as the means of all BMI and weight measures, respectively, reported across visits, with the missing values omitted.

Biological data often follow a log-normal distribution, as values of measured parameters cannot go below 0^(48)^. We adjusted the intake for body weight and applied a log-transformation to the data, discarding samples with no reported fibre intake and bringing the distribution close to normal. That led to another shrinkage of the dataset (final number of individuals between 19 987 and 19 990 depending on a particular type of fibre, see Table 2).

**Table 2:** Numbers of subjects in final reference groups used for calculation of reference values for different types of fibre

Fibre type	*n*	Men	Women
Soluble			
Soluble hemicelluloses	19 988	6940	13 048
Soluble pectin	19 897	6898	12 999
Insoluble			
Insoluble cellulose	19 990	6941	13 049
Insoluble hemicelluloses	19 990	6941	13 049
Insoluble pectin	19 980	6935	13 045
Insoluble lignin	19 987	6941	13 046
Total			
Total hemicelluloses	19 990	6941	13 049
Total pectin	19 980	6935	13 045
Total soluble fibre	19 988	6940	13 048
Total insoluble fibre	19 990	6941	13 049
Total fibre	19 990	6941	13 049

The reference ranges were calculated for 2·5th and 97·5th percentiles, which is the interval used most commonly in practice^(49)^. The CI for the reference range limits and the medians were calculated using the sampled data according to the method described by Hahn and Meeker^(50)^ and are guaranteed to be equal to or greater than 95 %. All the obtained values were then exponentiated to transfer them back into linear space.

Statistical analysis was performed using the Microsoft R Open programming language, version 3.5.2, and RStudio IDE, version 1.2·1335. *P*-value < 0·05 was considered statistically significant.

## Results

**Fig. 1:**
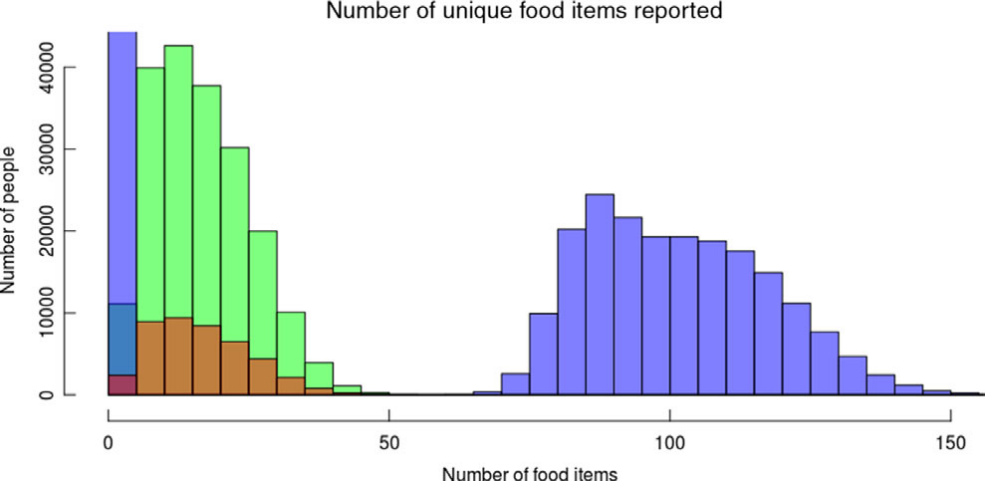
Counts of people reporting consumption of different numbers of foods at different stages of the sample preparation. 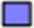, Before filtering; 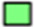, after filtering; 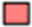, after filtering: only healthy

Shrinking the dataset was associated with an inevitable loss of fidelity, as illustrated in Fig. 1. As both the number of participants and the number of foods decrease, each food is reported less often, and the number of reported foods for each participant declines.

The association between body weight and the amount of ingested nutrients is typically accounted for in dietary studies. We performed the adjustment by dividing the fibre intake values by the corresponding body weight (prior to applying log-transformation).

**Fig. 2:**
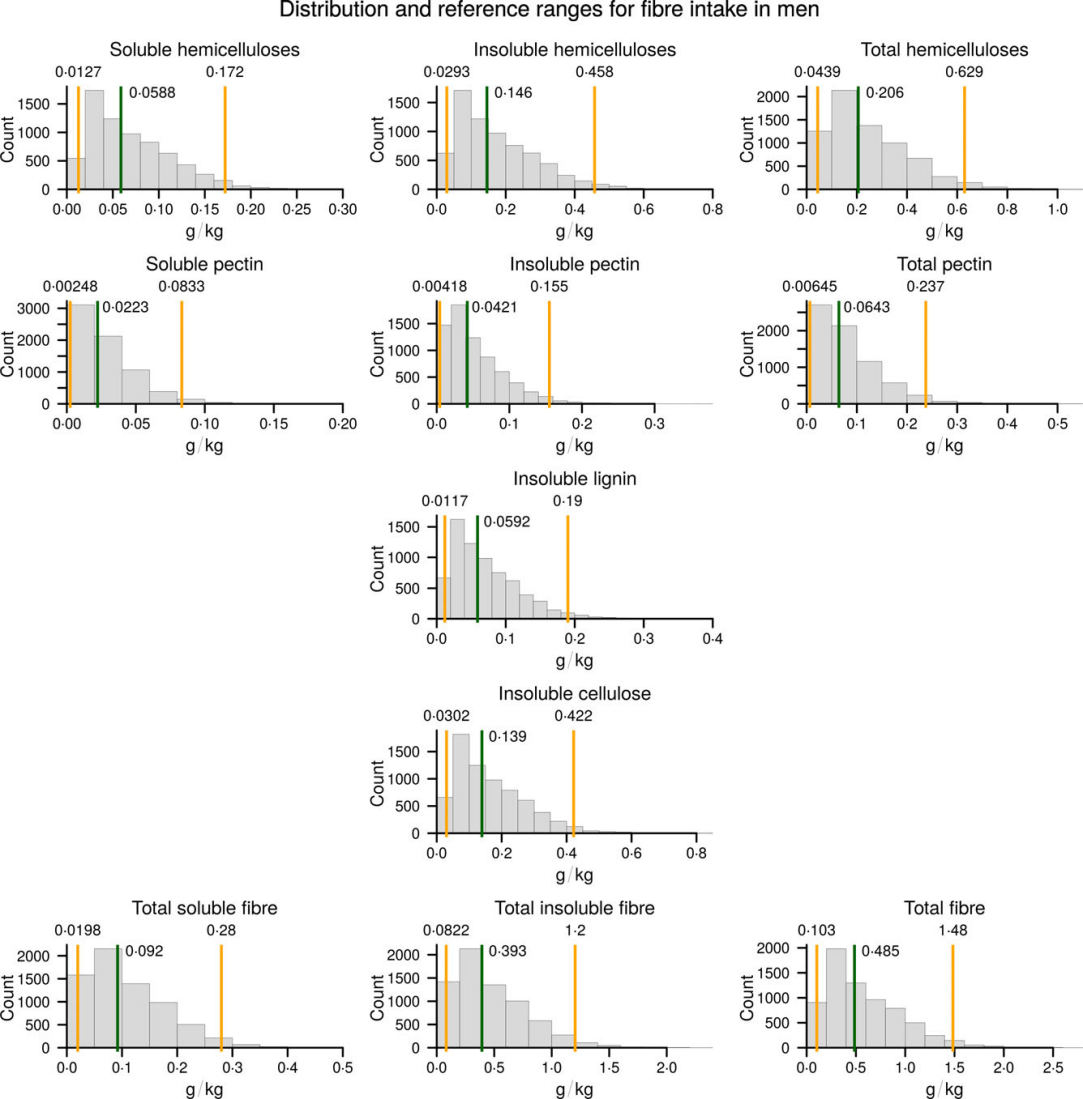
Consumption of different types of dietary fibre in a healthy male population. The calculated 2·5th and 97·5th percentiles are indicated by the orange vertical lines, and the calculated median is indicated by the green vertical line

**Fig. 3:**
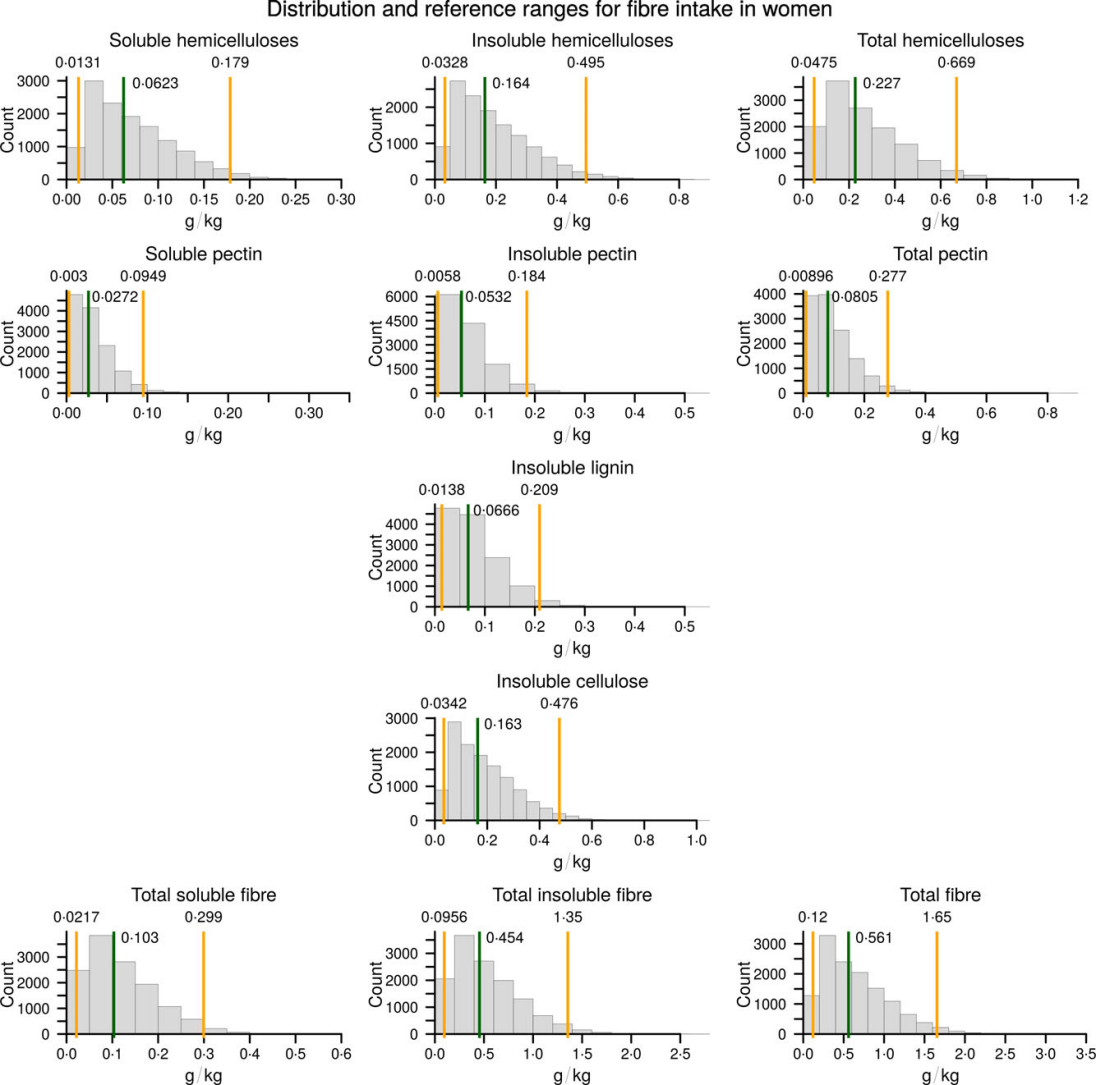
Consumption of different types of dietary fibre in a healthy female population. The calculated 2·5th and 97·5th percentiles are indicated by the orange vertical lines, and the calculated median is indicated by the green vertical line

The resulting reference values and the median values for the healthy population are presented in Table 3 as grams of fibre per kilogram of body weight. The graphical representations of population distributions of consumption for different types of fibre, as well as the reference and median values, are presented in Figs 2 and 3.

**Table 3:** A summary of the obtained reference values and medians for daily intake of different types of fibre, stratified by sex and fibre type

Fibre type	Daily reference intake, g/kg			
	Men		Women	
	Range	Median	Range	Median
Soluble hemicelluloses	0·013–0·172	0·059	0·013–0·179	0·062
Soluble pectin	0·002–0·083	0·022	0·003–0·095	0·027
Insoluble cellulose	0·03–0·422	0·139	0·034–0·476	0·163
Insoluble hemicelluloses	0·029–0·458	0·146	0·033–0·495	0·164
Insoluble pectin	0·004–0·155	0·042	0·006–0·184	0·053
Insoluble lignin	0·012–0·19	0·059	0·014–0·209	0·067
Total hemicelluloses	0·044–0·629	0·206	0·048–0·669	0·227
Total pectin	0·006–0·237	0·064	0·009–0·277	0·08
Total soluble fibre	0·02–0·28	0·092	0·022–0·299	0·103
Total insoluble fibre	0·082–1·203	0·393	0·096–1·354	0·454
Total fibre	0·103–1·484	0·485	0·12–1·654	0·561

## Discussion

### Analysis of the obtained results

The obtained results provide an estimated description of fibre consumption patterns in the healthy UK population. The final reported values have been adjusted for body weight.

According to the obtained reference values, insoluble fibre dominates over its soluble counterpart in typical diets. It is challenging to argue about the reason, as the insight into the specifics of the participants’ diets is limited. An intriguing explanation would be that it is caused by a specific dietary pattern which healthy individuals tend to adhere to. It is also plausible that food in general tends to contain more insoluble than soluble fibre, a fact which would inevitably affect the population statistic. Either way, this presents a new area of research which may be explored when a more robust dataset becomes available.

In the analysed cohort, fibre consumption per kilogram of body weight was slightly higher in women than in men for every type of analysed fibre. We propose two possible explanations for this fact. First of all, it has been shown that women tend to be more conscientious in their food choices, attaching greater importance to a ‘healthy’ diet^(51)^. Limiting the intake of high-fat and high-calorie foods is commonly associated with increased fibre intake, as fibre-rich foods tend to be less energy-dense^(3–5)^. Secondly, on average women tend to weigh less than men^(52)^, which would result in a higher intake per kilogram of body weight if the diets were identical.

### Analysis of methods in context of existing studies

Comparing the methods used in this study to the methods used in the similar epidemiological studies in the field of nutrition presents a challenging task, as there are, to our knowledge, no studies regarding the intake of fibre subtypes. It is, however, possible to compare the methodology of this study to the methods used in epidemiological studies of overall fibre intake. In this regard, it is necessary to look into the methods used for the estimation of dietary intake and for the quantification of fibre in the consumed food.

Twenty-four hour recall questionnaires seem to be a widely used tool in epidemiological research. This type of analysis has been used to estimate fibre intake in the US^(53)^ and Australian^(54)^ populations by nutrition researchers. In her review, Block^(55)^ states that, although insufficient to accurately analyse the diet of a single individual, 24-h recalls provide considerable insight into the dietary intake of a group of persons. The precision of this method is discussed by Bingham *et al.*, who conclude that a ‘24 recall method… compared surprisingly well with weighed records’^(56)^. This favourable account is further reinforced by Johansson^(57)^ who, in his study of different methods of food intake assessment, concluded that the foods reported by recording and recalling methods follow the same misreporting patterns and, therefore, the error is individual-specific and not method-specific.

The data on fibre content provided by Marlett and Cheung^(46)^ were obtained using the method A developed by Theander, more widely known as the Uppsala method^(58)^. In their analysis, Knudsen *et al.* ^(59)^ argue that this the precision of this method is primarily dependent on laboratory equipment and personnel experience, and that it has reached an acceptable level. Furthermore, they state that that the variation between the results obtained in different laboratories which use this method is comparable to the variation in the results obtained by other methods, such as the AOAC enzymatic-gravimetric methods and the Englyst methods.

### Analysis of results in context of existing studies

Comparing the obtained values to the existing reference values is also challenging, because the existing reference values do not account for specific types of fibre. Total fibre values from our study can be compared with the existing dietary guidelines; however, studies show that the dietary advice provided by the guidelines is rarely adhered to^(60–62)^, thus, such a comparison would introduce a certain degree of error.

A possible solution would be to use the ‘real-world’ values obtained in population studies for comparison. We used the work by Rippin *et al.* ^(60)^, which provides a weighted mean of fibre intake in twenty-one European countries of 19 and 21 g of fibre per d for women and men, respectively.

To calculate the ‘recommended’ intake for men and women using the values obtained in our study, we used the reference body weights provided by the Institute of Medicine^(52)^ (men: 70 kg, women: 57 kg). We multiplied these weights by the median of the total fibre consumption calculated in our study to obtain the ‘recommended’ fibre intake for these hypothetic persons. The resulting totals were compared to the values provided by the Institute of Medicine^(52)^ and by Rippin *et al.* ^(60)^ (Table 4). For convenience, we provide the reference ranges and medians for persons of this weight for all the analysed fibre types in Table 5.

**Table 4:** A comparison of recommended daily total intake of fibre between this study, Institute of Medicine,^(52)^ and Rippin *et al.* ^(60)^

Sex	Daily total fibre intake, g		
	This study, median	Institute of Medicine^(52)^	Rippin *et al.* ^(60)^
Male	33·95	30–38	21
Female	31·977	21–25	19

**Table 5:** The reference range and median daily intake of different types of fibre for a person of reference weight (70 kg for males and 57 kg for females)^(52)^, stratified by sex and fibre type

Fibre type	Daily reference intake, g			
	Male, 70 kg		Female, 57 kg	
	Range	Median	Range	Median
Soluble hemicelluloses	0·91–12·04	4·13	0·741–10·203	3·534
Soluble pectin	0·14–5·81	1·54	0·171–5·415	1·539
Insoluble cellulose	2·1–29·54	9·73	1·938–27·132	9·291
Insoluble hemicelluloses	2·03–32·06	10·22	1·881–28·215	9·348
Insoluble pectin	0·28–10·85	2·94	0·342–10·488	3·021
Insoluble lignin	0·84–13·3	4·13	0·798–11·913	3·819
Total hemicelluloses	3·08–44·03	14·42	2·736–38·133	12·939
Total pectin	0·42–16·59	4·48	0·513–15·789	4·56
Total soluble fibre	1·4–19·6	6·44	1·254–17·043	5·871
Total insoluble fibre	5·74–84·21	27·51	5·472–77·178	25·878
Total fibre	7·21–103·88	33·95	6·84–94·278	31·977

The result of our study corresponds to the reference values provided by the Institute of Medicine for individuals between 30 and 70 years old, although the reported values for women tend to be higher than expected. This difference is easily explainable: our calculation uses real-world consumption data, and it is logical that total consumption of fibre in men and women seems to be more uniform. The values provided by the Institute of Medicine^(52)^, on the contrary, represent a threshold to aim for, but not a precise snapshot of real-world consumption patterns.

The difference between our values and the real-world data reported by Rippin *et al.* ^(60)^ is more challenging to explain. One possible reason lies in the analytical techniques used for dataset preparation: some bias could have been introduced with removal of foods or participants. Another plausible explanation is the aggregate nature of the data produced by Rippin *et al.* ^(60)^: it encompasses many studies exploring different populations. Between-population variation of fibre intake would affect the weighted mean.

Fry *et al.* ^(63)^ found that the cohort of the UK Biobank is ‘healthier’ than the general population in terms of having less detrimental habits and chronic diseases. This fact is accounted for in the selection process of this study, as we implicitly removed the participants with any reported diseases to ensure that the analysed cohort consisted only of ‘completely healthy’ individuals. This ‘healthy bias’, however, could also stem from the certain dietary patterns in the analysed individuals; it is tempting to suggest that the better health of the UK Biobank cohort and the observed higher-than-average fibre intake are, in fact, interrelated. Further analysis would be required to confirm or disprove this hypothesis.

### A healthy diet: what is it, exactly?

Dietary fibre is a necessary component of a healthy diet^(64)^. Evidence shows that level of education may impact diet adherence; insufficient knowledge about nutrient content is among the most significant factors to influence a person’s decision to abandon a recommended regimen^(65–67)^. Conducting research into fibre seems to go hand in hand with promoting awareness about its importance, which can support public health initiatives as well as practitioners working with patients.

Different types of fibre exhibit different properties, so a balanced intake of various types of fibre is needed to satisfy the daily fibre requirement. Upsetting this balance may not only provide no benefit, but even become detrimental^(68)^.

However, this topic suffers from an alarming lack of clarity: the absence of reference values for specific types of fibre has been mentioned in literature, and such values were not available as of several years ago^(69)^. To the best of our knowledge, this is the first published study to estimate a reference intake range not for dietary fibre as a whole, but for its different soluble and insoluble subtypes in a population of healthy individuals. Hopefully, this article will lay the groundwork for developing this topic further.

### Dietary fibre and gut microbiota

Fibre serves as a substrate for beneficial bacteria to feed on^(39,70,71)^, and modifying its intake can shift microbial abundance and diversity. Non-digestible carbohydrates provide the primary source of energy for most gut microbes, and changes here impact bacterial communities that depend on particular fibre substrates ‘rapidly and reproducibly’^(72)^. Seemingly small increases in daily fibre content (as low as 6–8 g of wheat fibre per d) mediate changes to microbiota composition, species diversity, species abundance and metabolic indicators of microbiota fermentation such as SCFA or faecal nitrogen^(73)^. Changes in gut bacterial diversity and abundance correlate with improvements in cardiometabolic^(74–76)^, immune and inflammatory^(77–80)^ markers. In a series of systematic reviews and meta-analyses^(81)^, Reynolds *et al.* found that consumption of 25 to 29 g of fibre daily is associated with significant reductions in both mortality and incidence of a variety of pathological conditions. Similar intake is recommended by multiple other guidelines^(82,84)^.

There have been attempts to estimate the dietary fibre ‘preferences’ of different bacterial taxa. McKeown, Sawicki and colleagues used evidence mapping methodology, contributing to the creation of the Diet-Related Fibers & Human Health Outcomes Database^(84,85)^. Currently, quality evidence from randomised controlled trials is limited to the ability of the *Bifidobacterium* genus to ferment oligosaccharides, fructooligosaccharides in particular^(86–88)^.

McKeown and Sawicki also identified several methodological limitations in research on the effect of fibre subtypes on different types of gut microbes, including the use of diverse microbe identification and quantification methods. This lack of uniformity complicates the comparison of study results^(84,85)^.

Attempting to be more specific in matching bacterial genera with their preferred types of fibre thus remains an elusive task – not only because most foods provide a mix of soluble and insoluble fibres, but also due to other, more intricate, factors which impact microbial composition and abundance indirectly. One such factor is cross-feeding, a symbiotic relationship which enables certain microbes to survive by feeding on the metabolic byproducts of each other. This is seen in complex biological systems^(89)^ and particularly in the gut, where lactate produced by *Bifidobacteria* has been reported to stimulate the formation of butyrate by bacteria of other genera^(90,91)^. Another example is provided by butyrate-producing *Clostridiales*, a microbial order belonging to the *Firmicutes* phylum that are able to metabolise oligosaccharides in human milk and cross-feed on mucin via conserved pathways^(92)^.

Effects of cross-feeding are not always beneficial to the host. Hydrogenotrophic microbes (sulphate-reducing, acetogenic and methanogenic bacteria) are able to convert hydrogen into hydrogen sulphide, acetate and methane, respectively. Higher levels of these metabolites correlate with worse symptoms in irritable bowel syndrome, and other diseases of the gut^(93–97)^.

Some microbes in the *Lachnospiraceae* family, particularly the *Roseburia* genus, stand out in microbiome studies of Mediterranean diets, as does the *Faecalobacterium* genus. Specifically, the *Faecalobacterium prausnitzii* species^(98,99)^ can utilise pectin as a substrate for growth^(100)^. Both the *Roseburia* and the *Faecalobacterium* genera are known for their ability to ferment fibre, producing SCFA and other metabolites with bifidogenic properties^(101)^.

*Eubacterium* and *Coprococcus* genera share a similar behaviour and are often characteristic for people who consume diverse types of plant fibres^(102,105)^, alongside some members of the *Prevotella* genus^(105,105)^. Also observed in Mediterranean-style diets is a lesser abundance of microbes in the *Proteobacteria* phylum, particularly of *Enterobacteria* ^(106,107)^.

The availability of a specific feeding substrate is not the only factor influencing microbiota composition: stomach and small intestine pH, pancreatic and biliary function, transit time^(108–111)^, and even non-dietary psychosocial factors relating to mental health^(112–114)^ and levels of physical activity^(115)^ all play their role. These factors may affect the ratios and abundance of SCFA^(116)^, known to influence the composition of the microbiota via a decrease in colonic pH^(117,118)^.

It seems prudent to focus on overall changes in microbial diversity and composition associated with dietary patterns. ‘Mediterranean-type’ diets rich in varied types of fibre from brightly coloured fresh produce, legumes/pulses, wholegrains and oily fish are well known for their ability to influence microbiota. This dietary pattern is associated with positive health outcomes in a range of conditions^(119–122)^. Microbiome of individuals following a Mediterranean-style diets is highly abundant in *Bifidobacteria* and *Lactobacilli* ^(123–125)^.

### The role of gut microbiota in human health

As a community of microorganisms, the microbiota interacts with their human host through immune, neuroendocrine and neural pathways^(126)^, casting local and systemic effects on the host’s health and affecting their disease risks. These risks are modulated, in part, by fermenting non-digestible substrates such as dietary fibres^(127)^ and polyphenols^(128,129)^. This supports the growth of specialist microbes that produce SCFA^(130)^, as well as gases like methane and hydrogen^(131–133)^, further supporting the symbiotic relationship between microbial communities and the host. For instance, *Akkermansia muciniphila* ^(134)^, certain *Bacteroides* ^(135)^ and some *Bifidobacteria* ^(136)^ degrade the polysaccharides and highly glycosylated proteins present within the intestinal mucus^(137–139)^, supporting tissue barrier function^(140)^ and alleviating inflammation^(141,142)^.

Microbiota influences blood glucose homoeostasis and intestinal permeability and is associated with the modulation of gene expression in lipid and glucose uptake and transport pathways. Many of the effects are mediated by the production of butyrate by beneficial bacteria, which use prebiotic fibre present in food as an energy substrate. Such bacteria are depleted in fibre-poor dietary patterns such as the Western diet^(143,144)^.

Butyrate is the main source of energy for colonic epithelial cells; it contributes to healthy intestinal permeability^(145)^ and modulation of metabolic endotoxemia^(36,146,147)^. It has been shown that decreasing carbohydrate intake can lead to lower butyrate production in the colon of obese patients. Duncan *et al.* found that obese volunteers put on a 4-week diet of medium-carbohydrate intake, followed by 4 weeks on a low-carbohydrate diet showed a ‘disproportionate’ decrease in faecal butyrate and reduction in butyrate-producing bacteria^(148)^. Acetate and propionate, SCFA also produced by the colonic microbiota from prebiotic fibres, have been shown to participate in fat storage and appetite control. In addition, associations have been found between lean body mass and the presence of *Akkermansia muciniphila* ^(149)^, an acetate producer and mucin-degrading bacterial species whose activities stimulate the production of mucins in the mucosa, thus contributing to improved intestinal barrier function.

### Limitations and concerns

As a pilot study, this research has several shortcomings. Due to voluntary 24-h self-recall style of collection, the data could have been self-censored by the participants or could differ from their typical dietary pattern. The existence of the issue of self-censoring is indirectly confirmed by Bradbury *et al.* ^(150)^, who found that dietary findings were less reproducible in participants who had BMI > 25 than in those who had BMI < 25. Although this research only included individuals with BMI within a specific range, it is hard to estimate the degree of self-censorship their data had gone through.

The limited amount of data may have affected the distribution of fibre intake values. Limiting the number of analysed foods may have both decreased the reported fibre intake and skewed the distribution to the right. These effects would artificially lower the obtained reference values, which we have discussed earlier. However, the majority of foods excluded from the analysis were unlikely to contain fibre, as they were meat-, fish-, poultry- and dairy-based.

Applying a log-transformation meant that the individuals with no reported fibre intake had to be excluded from the analysis (logarithm of 0 is undefined). However, these individuals might constitute a significant portion of the population, which means that our approach resulted in an increase in the obtained values. This may offset the decrease described earlier.

The difference between the 2·5th and 97·5th percentiles can be increasingly large, sometimes even reaching the order of several magnitudes. This may be explained by the right skew of the data, which results in the increased value of the 97·5th percentile. This is especially apparent when reference ranges are calculated using specific body mass, as in Table 5. A possible solution would be to obtain higher-quality data on fibre content, preventing the exclusion of certain foods and certain individuals, thus decreasing the skew. An alternative solution would be to resort to using other, less canonical, values to limit the reference range, such as the 90th percentile, the 3rd quartile or even the median.

The nature of UK Biobank limited the age range of the subjects analysed in this study. Apart from the that, a typical area of concern is the low response rate and the existence of selection bias due to the volunteer-based nature of the cohort. Naturally, this raises concerns regarding the generalisability of the obtained results^(151)^. Despite our best efforts, we could not find any evidence regarding the validity of generalisations in the context of the 24-h recall questionnaire used to assess food intake. However, there are several studies that explore the representativeness of the analysed cohort and generalisability of the results regarding risk factor profiles. Perhaps, the most notable is the study by Batty *et al.* ^(152)^, who compared the risk factor profiles obtained using the UK Biobank to the risk profiles obtained using 18 cohort studies of English and Scottish populations. The authors found that, despite the low response rate of the UK Biobank participants, the data were comparable and concluded that the data obtained from the UK Biobank are generalisable to England and Scotland.

The most substantial issue that may have influenced the accuracy of the calculated values is associated with food mapping. Insufficient data on portion size of UK Biobank foods and their fibre content were available, and to merge UK Biobank data with the selected reference literature^(46,47)^ we had to use multiple generalisations. It has been shown that in certain cases substitutions may negatively affect the results even in closely related foods^(153)^. It would be intriguing to perform further similar analyses with datasets of improved quality, as it may improve the precision of the obtained result.

Insufficient data on portion size of UK Biobank foods and their fibre content were available. Therefore, in order to merge UK Biobank data with the selected reference literature^(46,47)^, we had to use multiple generalisations. Our primary concerns were about the validity of Marlett and Cheung’s food composition database, chiefly about the accuracy of a resource that is representative of the food supply in the USA. However, upon a thorough review of the literature, we were not able to identify major differences in fibre content among diverse varieties of fruit, vegetables or wholegrains. As a recent example, Koutsos *et al.* ^(154)^ performed nutrient composition analysis of three commercial apple varieties available worldwide: Renetta Canada, Golden Delicious and Pink Lady and found negligible differences in total dietary fibre amongst them, 2·6, 2·4 and 2·6 g/100 g (AOAC), respectively. Additionally, a comparative analysis of the food composition table (Tabela da Composição de Alimentos) published by the Portuguese National Health Institute and the US Department of Agriculture FoodData database carried out by Delgado *et al.* ^(155)^ found no discernible differences in the total fibre content of a range of foods typically available in the UK, including garlic, onions, cabbage, turnips, lettuce, tomato, pumpkin, wild greens such as watercress, and herbs like parsley, oregano or coriander. The authors did not find any major differences in the total fibre content of different varieties of wheat, rye, rice, potatoes or pulses such as beans, lentils or chickpeas. On the basis of the arguments laid out above, we are reassured that it is unlikely that any significant differences in fibre content would be detectable in samples of different varieties of the same food obtained from different geographic regions.

We must highlight some additional caveats to the quantification of total fibre content. One relies on whether a fruit or vegetable is peeled or not. For example, potato peels are known sources of dietary fibre, so much so that it doubles if the peel is consumed^(156)^. Another important consideration is grouping of foods. As an example, green peas have very similar amount of dietary fibre to pulses, and a significant portion of their starch is digested in the large intestine, providing substrate for colonic bacteria^(157)^. Furthermore, an increasing number of consumer goods containing added fibre are launched every year, making it difficult to develop a wholly comprehensive database of fibre values that is always up to date.

**Fig. 4:**
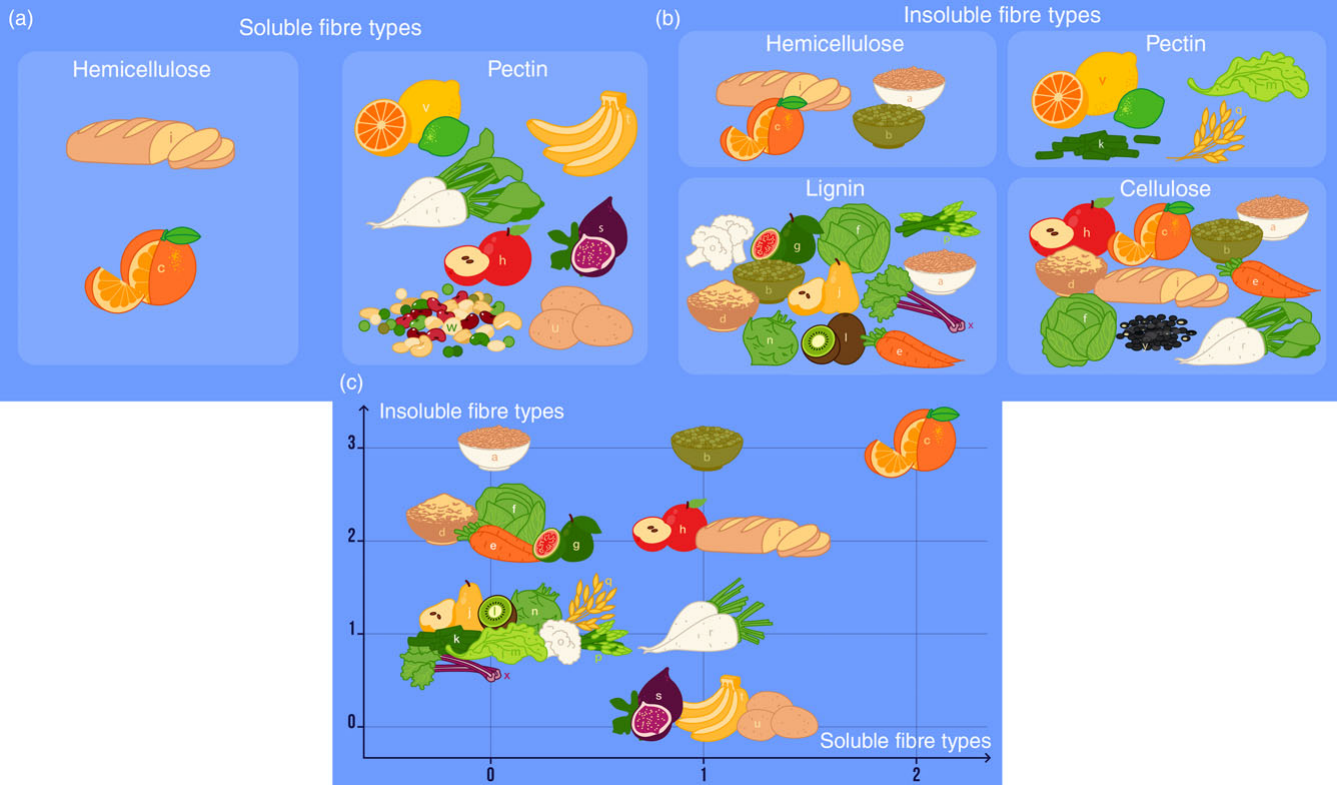
Quick-reference visualisation of abundance of different types of fibre in various common foods: (a) foods rich in soluble fibre; (b) foods rich in insoluble fibre and (c) food by number of fibre types. Different food items are coded as follows: a – rice bran; b – lentils; c – oranges; d – wheat bran; e – carrots; f – cabbages; g – guava; h – apples; i – white bread; j – pears; k – green beans; l – kiwi; m – lettuce; n – kohlrabi; o – cauliflower; p – asparagus; q – cereal grains; r – sugar beets; s – figs; t – bananas; u – potatoes; v – black gram; w – legumes; x – rhubarb

Despite these concerns, we believe that this study may not only serve as a primer for research into consumption of types of fibre, but also be used as a helpful guide when planning dietary interventions. To further increase its usability, we provide several easy-to-use diagrams for quick reference (Fig. 4). Further areas of research on this topic may include refining the obtained data and increasing their precision or exploring the association between the consumption of certain types of fibre and subjective and objective outcomes, such as development of certain diseases and quality of life. Research should also be aimed at compiling more comprehensive datasets on fibre content of foods, which in turn would provide the basis for a more detailed and precise analysis.

Based on our discussion, we propose a sample menu (Table 6) as a realistic and sustainable example of how to incorporate the amounts of soluble and insoluble fibres recommended in our research. The foods featured in this meal is presented visually in Figs 4(a), (b) and (c). This menu can be used as a sample to build other dietary options upon, or as a ready solution to be incorporated into the individual’s meal plan.

**Table 6:** A sample 1-d menu designed to introduce a recommended amount of fibre subtypes discussed in the article

Meal	Food	Quantity, g	Fibre content, g						
			Soluble hemicelluloses	Soluble pectin	Cellulose	Insoluble hemicelluloses	Insoluble pectin	Lignin	Total
Breakfast	All bran cereal	60 g	0·7	Trace	0	0	0	0	10
	Banana, sliced	120 g	0·3	0·3	0·4	0·3	0·3	0·7	2
Snack	Apple	130 g	0·2	0·3	1·1	0·9	0·6	0·4	3·1
Lunch	Baked beans	150 g	0·8	0·3	9·6	5·4	0·8	5·8	6·8
	Wholemeal toast (2 slices)	70 g	0·7	0	1·2	2·4	Trace	0·8	4·7
Dinner	Baked potato with skin, tuna mayonnaise	180 g	0·4	0·4	0·9	0·7	0·3	0·3	6·5
	Salad (lettuce, tomato and cucumber)	138 g	0·3	0·4	0·3	0·3	0·3	0·9	1·7
	Yogurt	150 g	0	0	0	0	0	0	0
	with strawberries	100 g	0·2	0·4	0·5	0·4	0·7	0	1·5
	and chopped almonds	13 g	Trace	Trace	0·4	0·8	0·4	0·5	1·3
Total			4·2	2·1	14·4	10·2	3·4	9·4	37·6

## Conclusion

We calculated reference intake ranges for six different types of soluble (hemicelluloses and pectin) and insoluble (cellulose, hemicelluloses, pectin and lignin) fibre, as well as the corresponding totals, for a healthy UK cohort of approximately 20 000 participants of the UK Biobank. As per standard protocols, we used the 2·5th and the 97·5th percentiles of daily intake as the lower and the upper bounds for the reference range (Table 3). The absolute values of reference ranges were then calculated using the median body mass provided by the Institute of Medicine^(52)^ (Table 5). Comparable results were obtained for men and women, with the tendency for values in men to be slightly larger. A graphical summary of fibre content in different foods has been developed for practical convenience (Figs 4(a)–(c)), and a sample menu has been composed to introduce a balanced fibre intake.
